# Water Ecosystem Services in Northern Australia—How Much Are They Worth and Who Should Pay for Their Provision?

**DOI:** 10.1371/journal.pone.0064411

**Published:** 2013-05-24

**Authors:** Kerstin K. Zander, Rowena Parkes, Anna Straton, Stephen T. Garnett

**Affiliations:** 1 The Northern Institute, Charles Darwin University, Darwin, Northern Territory, Australia; 2 School of Health, Business and Science, Batchelor Institute of Indigenous Tertiary Education, Katherine, Northern Territory, Australia; 3 Victorian Department of Primary Industries, Melbourne, Victoria, Australia; 4 Research Institute for the Environment and Livelihoods, Charles Darwin University, Darwin, Northern Territory, Australia; University of Florida, United States of America

## Abstract

There is ongoing pressure to develop the largely unaltered Daly River catchment in northern Australia for agriculture. However, a choice experiment among people in the region and in Australia’s largest city, Sydney, shows that people are prepared to pay substantial amounts to maintain the quality of its ecosystem services. The total stated willingness-to-pay (WTP) for a Daly River conservation programme was about $300, of which people would be willing to pay over half ($161) if the programme retained waterholes for Aboriginal people in good condition. The WTP for high quality recreational fishing and biodiversity values was $120 and $91 respectively. Using the average cost of a recreational fishing license in Australia ($35) as a basis for grounding the stated preferences in empirical values, as well as the cost of park entry fees and the amount of support society provides to agriculture in Australia, the total amount that the 110,000 people in the region are likely to be willing to pay for the retention of the values in the Daly River catchment is about $6 million, while the 4.5 million people in Sydney would be willing to pay about $81 million. A significant finding in this research is that, while fishing, biodiversity and agricultural values all have equivalents in the market economy, the value for which people were willing to pay most, the cultural value, has no equivalent at all and is thus receives almost no investment.

## Introduction

In recent decades, market-based approaches to conservation management have become increasingly important, of which the best known is Payments for Environmental Services (PES). PES schemes are direct payments conditional on the services provided, i.e. they are based on incentives. PES can be more cost-effective than unconditional and/or indirect payments [1] and, while mostly designed to provide natural resource management, they can, under special circumstances, contribute to livelihoods and alleviate poverty [2]. In theory, under PES, the external ecosystem services beneficiaries make conditional payments directly to those who provide well-defined services [3]. In practice, these two issues are not so straightforward. First of all, if the full range of beneficiaries is unknown, it cannot be guaranteed that the ‘right’ people pay, including many distant users who benefit the most but do not pay [4]. Secondly, it is often not possible to measure the biophysical outcome of services provided, because of non-standardized methods and lack of concise classifications and definitions of ecosystem services [5], [6], but what can be measured is the benefit these services have to the society. Fisher et al. [7] argue that functions and processes of ecosystems only become services when there are human beneficiaries.

For more than a decade economists have tried to incorporate environmental goods and services into the market model [8] by introducing market-based instruments such as PES, thereby aiming to “internalize external benefits” [9], i.e. decreasing the burden to local users who bear the costs of ecosystem service provision. We argue, contra Wunder [3], that this can be achieved by proper economic valuation of the benefits provided by ecosystem services, measured by the values beneficiaries express for them not only to local users but to non-user beneficiaries who value the potential or existence value of the services. The Millennium Ecosystem Assessment [10] and the ‘The Economics of Ecosystems and Biodiversity’ (TEEB) report [11] support this view and highlight the need for increased research on measuring ecosystem services and assessing changes in their provision with respect to human welfare.

The tropical river systems of northern Australia are still relatively intact compared to Australia’s temperate river systems, carrying about 70% of Australia’s freshwater runoff [12]. One of the largest, and biologically most diverse, free-flowing rivers in northern Australia is the Daly River of the Northern Territory (NT). It provides many ecosystem services for the wellbeing of people and industries such as pastoralism and horticulture while its associated wetlands and estuaries are nationally and internationally recognized for their ecological and cultural values. However the relative importance of these different values is poorly understood. Ongoing droughts and increasing water demand in southern Australia has emphasized the potential for further development of tropical rivers for pastoral land and irrigated agriculture, putting pressure on the intact systems of the Daly River [13]. There is also a need for active management of existing ecosystems to reduce weed incursions, control feral animals and maintain a healthy fire regime. Thus conservation planning for the Daly River is necessary to secure the supply of ecosystem services into the future. Evidence of the relative value of the river’s multiple ecosystem services can have an impact on alternative scenarios for water-resources management while understanding who benefits from the rivers’ ecosystem services is important for targeting conservation investments.

The aim of this paper is to quantify the values and benefits of ecosystem services provided by the Daly River to help estimate the amount that could be paid to those who can, and would like to, maintain these services. The ecosystem services are split into recreational, cultural, environmental and productive services and their values are assessed through a survey-based stated preference method. We also assess whether benefits of these services are perceived differently by people who live close to the river, and so readily benefit, compared with distant potential users.

### Valuation of Environmental Services

Ecologists assess ecosystem services provided in terms of units per hectare of land or water [14], the ecological processes involved in service provision and interrelationships among services. Economists on the other hand assess the benefits of ecosystem services by determining who the beneficiaries are and the worth of the benefits derived by those people. A common approach for the last 40 years, and sometimes the only way of providing economic value of environmental goods, is to estimate people’s willingness-to-pay (WTP) for these goods [15]. One of the more sophisticated methods, choice experiments (CE), is now starting to be applied to revealing the value for ecosystem services to people based on their stated preferences in hypothetical situations [16], [17], [18]. In Australia, this method has been used extensively to assess river ecosystems [19], [20], [21], [22], revealing people’s stated WTP for favorable ecosystem services and their willingness-to-accept (WTA) compensation for unfavorable ecosystem services. The net sum of all WTP and WTA estimates constitutes the Total Economic Value (TEV) of a conservation program that maintains the ecosystem services. The TEV includes not only values that are reflected in markets via price mechanisms but also intangible non-market values. The TEV of ecosystem service conservation can be broadly classified into use (including direct, indirect or optional values) and non-use values (including existence and bequest values). The optional value is often found to encompass both use and non-use values, as it reflects the WTP to maintain an environmental good/service in existence in order to preserve the option of using it in the future [23].

Assessing the TEV of an ecosystem service conservation program allows the services’ proper incorporation into formal markets as public goods which are often underestimated and under-provided. It is also necessary for conducting cost-benefit analyses, assessing the benefits of development versus the costs of disfunctional services. This may help policy-makers by providing economic arguments for conservation [15]. Most development versus conservation decisions are based on market prices, but for many ecosystem services no markets exist, and decision-makers have no proxy for the value of the services [11], [24], a fact which disguises the TEV of the services. This is considered one of the main reasons for the decline of ecosystem services [10].

From an ecological point of view, a healthy river system provides many ecosystem services. These include provisioning services (e.g. food supply, oxygen production, provision of genetic resources), regulating services (e.g. climate regulation, waste treatment, biological control) and supporting services (e.g. support for primary production (irrigation), nutrient cycling, species diversity maintenance) [25]. In order to carry out economic evaluation, these ecosystem services have to be classified in such a way that their benefits to human society can be measured. We transformed the Daly River’s ecosystem services into 1) environmental, 2) recreational, 3) cultural and 4) productive ecosystem services (Table 1). Each ecosystem service is a component of the river’s TEV and each can be managed to ensure the service is conserved (Table 1: far right column).

**Table 1:** Attributes and level used in the choice experiment.

Attribute	Levels+	Service	Component of TEV++
Area of floodplain in good environmental condition	**Small**	Environmental	Indirect/Direct use-value
	Medium		Option value
	Large		Existence value
Quality of the river for recreational fishing	**1-Star**	Recreational	Indirect/Direct use-value
	3-Stars		
	4-Stars		Option value
Conditions of waterholes important to Aboriginal people	**Poor**	Cultural	Direct use value
	Ok		
	Good		Existence value
Income from irrigated agriculture	Low	Productive	Direct use value
	Medium		
	**High**		
Cost of management plan (in AUS$)	**0**		
	10		
	50		
	100		

+ the *status-quo* levels are indicated in bold.

++ TEV: Total Economic Value.

## Methods

### The Case Study

The Daly River catchment area is located approximately 200 km south of Darwin and covers an area of approximately 52,500 km2 (as big as Costa Rica). Several major rivers, including the Katherine, Dry, Flora, Fergusson, Douglas, Fish, join to form the Daly River which flows west into the Timor Sea. The population of the Daly River catchment area is approximately 10,000 people at a density of less than 0.2 per km2. Twenty-eight percent of the population is Aboriginal and 15% of the catchment is under Aboriginal freehold title. For Aboriginal people the river holds particularly high cultural significance [26], [27]. The floodplains of the river have very high biodiversity value, in some years supporting up to 36% of the population of the iconic Magpie Goose *Anseranas semipalmata* [28], but the health of the habitat is correlated with river flow, so would be affected if more water is extracted for irrigation, and the control of invasive exotic weeds [29]. Around 60% of the land is managed for cattle of which 5% has been cleared to create pasture. Less than 1% is irrigated, drawing 76,000 Ml per year [30]. Other important industries include fishing and aquaculture, tourism and recreation, defense and mining. Water is critical to many of these industries and future expansion of the mentioned industries might generate increased competition amongst water uses and users.

### Sampling and Survey Mode

**Figure 1:**
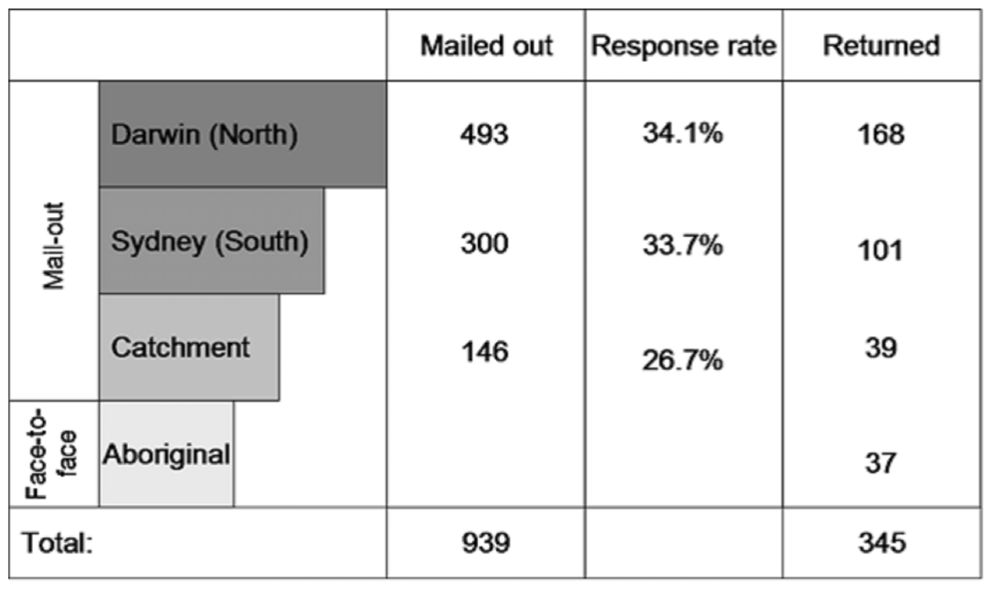
Sampling and response rates.

Two populations were sampled: people in the northern NT (Darwin and the Daly River catchment area; population 150,000) and people from southern Australia (Sydney; population 4.5 m), nearly 4,000 km from the Daly River catchment. The urban centres Darwin and Sydney were chosen to represent local and distant urban users. First an introductory letter was sent and after two weeks the questionnaire. Respondents were randomly chosen from the White Pages and the introductory letter was sent to 1,100 people, of which 14.6% were undeliverable and returned to sender. The survey was then mailed out to the remainder: 300 to Sydney, 493 to Darwin citizens and 183 to the catchment residents (Fig. 1). Each mailing included a personally signed covering letter, the questionnaire, a map indicating the location of the Daly River catchment, a freepost return envelope with a real stamp and a $1 coin incentive. Ethic approval for surveys with humans was obtained from the Commonwealth Scientific and Industrial Research Organization (CSIRO).

Additionally, 37 Aboriginal people, as representatives of traditional owners of catchment lands, were interviewed in the catchment area by the Aboriginal member of the research team. The face-to-face survey mode was employed with Aboriginal people because of language and cultural barriers. The questions and the CE were the same but the questionnaires were explained individually by the researchers in English or an appropriate Aboriginal language.

### Survey Design and Administration

The questionnaires had four main sections: 1) questions aimed at respondents’ attitudes towards environmental issues, 2) the choice questions beginning with an explanation of the attributes and the hypothetical scenarios, 3) follow-up questions to the choice questions to determine respondents’ motivations for their choices, and 4) questions on socioeconomic characteristics. For the CE, a primary list of attributes and levels was obtained after focus-group discussions and in-depth interviews with relevant stakeholders, including Aboriginal people. After a pilot study with 30 respondents had been conducted in Darwin, this primary list was slightly modified and final attributes and levels were defined (Table 1). One of these attributes was monetary (a one-off voluntary payment) so that WTP estimates for changes in attribute levels could be obtained.

**Figure 2:**
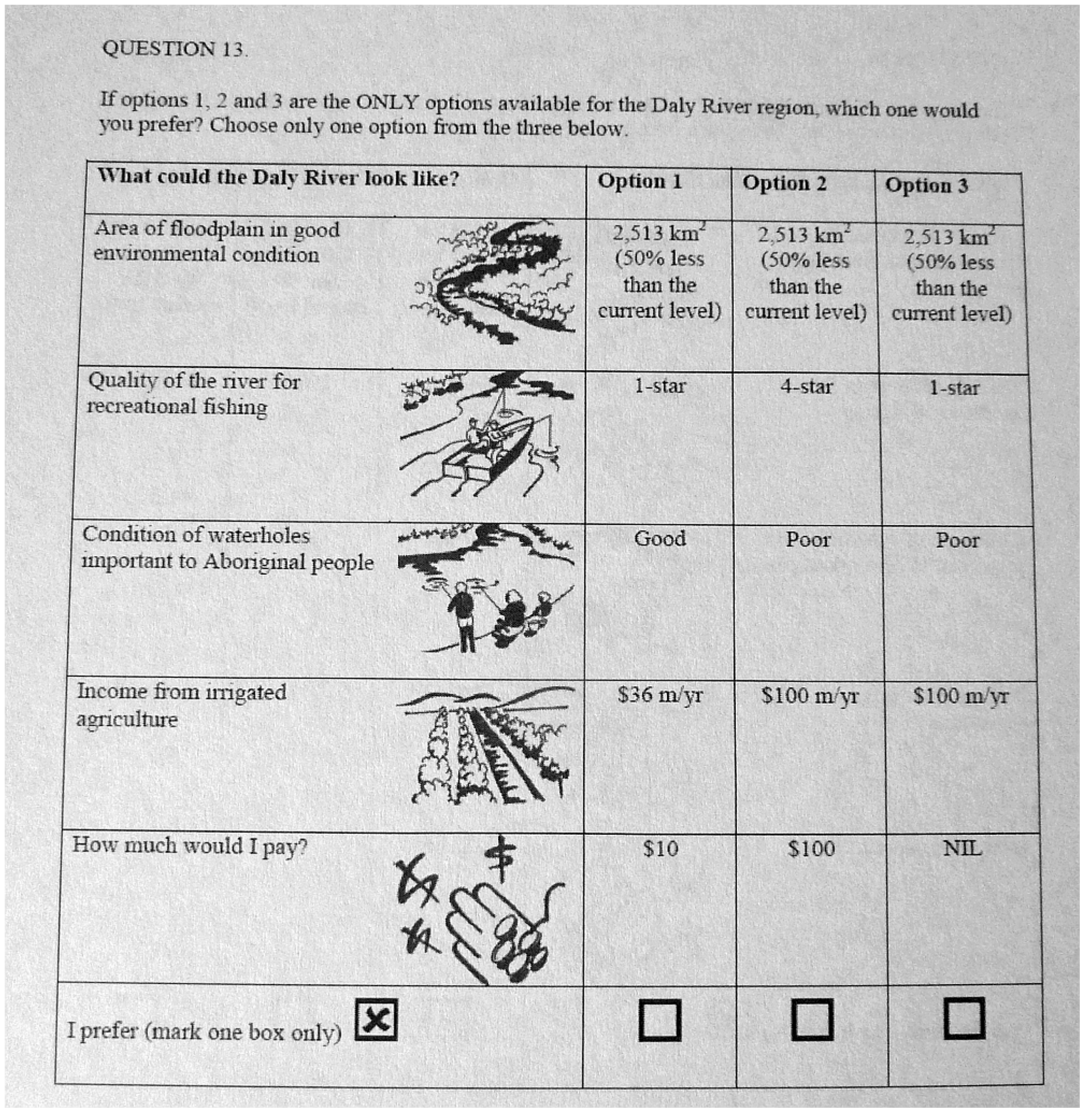
Example of a choice card used in the survey.

The final choice sets included three hypothetical scenarios from which respondents would choose their preferred one (Fig. 2). The associated question read: “If these three are the ONLY options available for the Daly River region, which one would you want to see?” Options 1 and 2 described outcomes of a conservation program for the Daly River. These two options had a cost associated with supporting the conservation program and ensuring the levels of the ecosystem service did not decline. The third option described the current situation, the *status-quo* (SQ), of conservation efforts for the Daly River. This SQ option represented a policy in which no additional money was made available for conservation, but instead development would be pushed forward, expressed by more income from irrigated agriculture. The ecosystem services were described under the SQ option as poor compared to the other two options.

The way in which the selected attributes and levels are combined into the above mentioned options is a fundamental part of stated choice modeling. The design of our experiment was based on the D-efficiency criterion, aiming to maximize the expected precision of the parameter estimates [31]. We used prior knowledge of the parameters to improve the design further [30]. For this Bayesian efficient design [32] we relied on priors from similar studies [19], [33], [34] as a proxy for the expected signs of coefficients. The final design yielded 24 choice sets which were divided into three blocks: two with eight choice sets and one with seven. One choice set was dismissed from the full set because it was behaviorally unrealistic. Each respondent was provided with one of the blocks and so answered either eight or seven choice questions.

### Analysis

The choice data were analyzed using a random parameter logit (RPL) model. This model is highly flexible [35] and is often used in studies that are concerned with revealing patterns of taste heterogeneity by allowing each attribute’s coefficient to vary over respondents [36] assuming a specific distribution [35]. We accounted for the fact that not all respondents attended to all attributes when making their choices by restricting these respondents’ parameters for the ignored attributes to zero [37], [38]. By constraining them to zero we assumed that respondents would have zero value for the ignored attributes. This approach has commonly been applied when dealing with non-attended attributes and has been found to provide more realistic WTP estimates as well as better model fits [37], [39].

The model coefficients are used to calculate respondents’ stated monetary values for the provided benefits of water services, expressed as their WTP or WTA compensation. These are estimates representing marginal changes in the level of provision of each ecosystem service (attribute in the experiment). In our case, they express discrete changes in an attribute level relative to the SQ level and thus provide information on the relative importance that respondents assign to the attributes used in the choice experiment. The monetary values and 95% confidence intervals were simulated from the chosen distribution using parametric bootstrapping [40], estimating a distribution of 10,000 observations for each value. We used dummy coding (0/1) for all attributes except the monetary attribute which was linear coded as the actual AUS$ amounts. All attributes had three levels (Table 1) and for each attribute we created two dummy-coded variables for the non-SQ levels. For the attribute ‘fishing quality’, for instance, we created the variables ‘3-stars fishing quality’ and ‘4-stars fishing quality’ both of which could be either one or zero. The models will yield coefficients for these two variables, both relative to the SQ level which was ‘1-star fishing quality’.

### The Aggregation of Benefits

Cost-benefit analysis requires the aggregation of benefit value estimates for comparison with the total costs of a project or policy [41], [42]. For a Daly River conservation programme which results in the highest levels of environmental quality, including a large area of healthy floodplains, 4-star fishing quality, waterholes in good condition and low income from irrigated agriculture, we compared the aggregated benefits from a policy resulting in these outcomes to one with low or no conservation management and hence low levels of environmental quality. We did this by multiplying the sum of the mean monetary values for all relevant services for a conservation scenario by the relevant population in the jurisdictions. Distance-decay functions [41], [43] could not be used because we lacked information about respondents’ locations other than the division into North and South. One of the most important concerns in aggregating values is to define the relevant aggregation population, i.e. finding those people who would benefit from the river’s ecosystem services. We defined the aggregation population as the number of individuals in the labour force (so able to pay) within three geographical boundaries, including: 1) individuals living in the north (within the Daly River catchment area and Darwin), 2) individuals in Sydney, and 3) all Australians. Although only 4% of respondents to the survey said they would pay nothing for the river’s conservation, we conservatively accounted for non-payers in the relevant population in each geographical jurisdiction by assuming that 70% of those who did not return the questionnaire (67%) would not be willing to pay, based on Morrison’s [42] approach.

## Results

### Respondents’ Characteristics

Some of the 345 returned questionnaires (see Fig. 1) could not be used for data analysis because none of the choice questions were filled out (4), respondents stated that they had not understood the choice questions (6), they considered none of the five attributes in a follow-up question (3), or they only attended to the cost attribute and no other attribute (10). From the 322 valid responses, 224 were from the North (Darwin: 156, catchment face-to-face: 37, catchment mail-out: 31) and 98 from southern Australia (Sydney). The sample included slightly more male than female respondents (52% to 48% in the whole sample; 57% to 43% in southern Australia; 50% to 50% in the North). The mean age was 52 (range: 16–87) without significant differences between respondents from the North and the South. Only 4% of respondents (4 in the South and 8 in the North) selected the SQ option in all presented choice questions.

### Respondents’ Importance of Attributes for Making Choices

A self-stating follow up question after all choice questions revealed that not all respondents considered all five attributes when making their choices. In fact, only 11% of respondents considered all the attributes, with no significant difference between respondents from northern and southern Australia. Most respondents (41%) considered three out of the five attributes, followed by two attributes (22%). Those ten respondents who only looked at the cost attribute were deleted from the sample because they made no trade-offs between conservation benefits and costs and hence these respondent’s marginal values for attributes could not be calculated.

More than three quarters of respondents (78%) considered the attribute ‘Condition of floodplains’ (Table 2) and 65% the ‘Quality of recreational fishing’ and ‘Condition of waterholes’. Less than half (43%) considered ‘Income from irrigated agriculture’ and only 29% the costs of management. Respondents from southern Australia considered the attributes ‘Condition of waterholes’ and ‘Income from irrigated agriculture’ more frequently (78% to 59% and 53% to 38%, respectively) than respondents from the North. Respondents from the North looked more frequently at ‘Quality of recreational fishing’ when making their choices (69% to 53%).

**Table 2:** Percentage of respondents stated to have attended to an attribute.

Attribute	Australia	Northern Australia	Southern Australia
Condition of floodplains	78%	76%	82%
Quality of recreational fishing	65%	69%	53%
Conditions of waterholes	65%	59%	78%
Income from irrigated agriculture	43%	38%	53%
Cost of management plan	29%	26%	36%

### Results of the Choice Experiment

A variety of RPL models were estimated with various distributions of parameters (normal, lognormal), including heterogeneity in means and variances of these distributions with respect to individual-specific socio-economic data. In addition, we tested models with error components included. Since each respondent faced a series of choice-sets, a panel data specification of errors was used throughout. We included an alternative specific constant for the SQ option throughout the analysis, although the attributes are generic, because it captures effects of unobserved sources of utility, leading to biased attribute parameter estimates [44]. The best fitting model was found to be one with all attributes included as random parameters with a normal distribution except for the cost attribute. We deliberately used a constrained triangular distribution for the cost attribute to avoid a change in signs and to ensure a negative sign of the cost attribute across all respondents [45], [46]. Maximum likelihood procedures were used to estimate the parameters of the choice model using the software Nlogit and all models were estimated with 500 Halton draws.

In all three samples the cost attribute had the expected negative sign and all other attributes the expected positive signs relative to the SQ option (Table 3). All attributes were statistically significant below the 1% level except for ‘medium income from irrigated agriculture’ which was insignificant. Hence respondents did not distinguish between the medium and the SQ level (‘high income from irrigated agriculture’), but disliked low income compared to the two higher levels. The constant for the SQ option was significant and negative for two of the samples, signifying that respondents were more likely to choose a river management option rather than the SQ option. Almost all standard deviations for the parameters of the random parameter estimates were statistically significant at the 1% level, suggesting that there was a great preference variation across respondents (Table 3). Consideration of the important policy implications of this variation is provided in Zander and Straton [27] and Zander et al. [20]. Here we investigate preference variation, and hence variation in monetary values across local respondents from the North and the distant respondents from southern Australia, by running separate models for the two sub-samples.

**Table 3:** Panel-RPL model results for the entire sample, north Australian sample and southern Australia sample.

	Australia			Northern Australia			Southern Australia	
Variable	Coeff.		SE+	Coeff.		SE+	Coeff.	SE+
Healthy floodplains medium	0.799***		0.170	0.714***		0.208	0.845***	0.291
Healthy floodplains large	1.864***		0.193	2.003***		0.242	1.495***	0.266
3-star fishing quality	2.042***		0.261	2.099***		0.325	1.889***	0.486
4-star fishing quality	2.460***		0.212	2.606***		0.282	2.587***	0.409
Waterholes in ok condition	2.386***		0.377	1.570***		0.414	3.203***	0.586
Waterholes in good condition	3.301***		0.261	3.325***		0.358	3.638***	0.447
Low income from irrigated agriculture	−1.559***		0.237	−1.521***		0.330	−1.848***	0.339
Medium income from irrigated agriculture	0.237		0.353	0.161		0.403	−0.257	0.551
Costs	−0.021***		0.003	−0.754***		0.165	−0.035***	0.006
SQ	−0.753***		0.139	0.714***		0.208	−1.072***	0.283
	Standard deviation of parameter distributions							
Healthy floodplains medium (normal)	0.899***		0.226	0.961***		0.266	0.839*	0.438
Healthy floodplains large (normal)	1.434***		0.194	1.475***		0.233	0.753**	0.355
3-star fishing quality (normal)	1.440***		0.371	1.855***		0.428	0.266	0.932
4-star fishing quality (normal)	1.789***		0.209	2.099***		0.271	1.562***	0.383
Waterholes in ok condition (normal)	1.688***		0.374	1.616***		0.519	0.936	1.296
Waterholes in good condition (normal)	2.205***		0.276	2.748***		0.358	0.916**	0.418
Low income from irrigated agriculture (normal)	1.724***		0.246	2.112***		0.363	1.061***	0.399
Medium income from irrigated agriculture (normal)	1.721***		0.351	1.693***		0.425	1.781***	0.639
Costs (triangular)	0.021***		0.003	0.016***		0.003	0.035***	0.006
		**Model fit**						
Log likelihood function	−1504.38				−1072.97		−403.12	
McFadden Pseudo R^2^	0.43				0.42		0.50	
Number of observations	2418				1685		733	
Number of respondents	322				224		98	
Halton draws	500				500		500	

*** 1% significance level; ** = 5% significance level; * = 10% significance level.

+ SE: Standard error.

Individual monetary values for changes relative to the SQ levels and their 95% confidence intervals (Table 4) indicate that respondents were willing to pay the most for ‘waterholes in good condition’ ($161 overall; $103 for respondents from southern Australia and $207 for respondents from the North). Respondents from the North were willing to pay substantially more for ‘large area of healthy floodplains’ ($125) and for the fishing quality ($131 for ‘3-stars’ and $163 for ‘4-stars’) than respondents from southern Australia. This may reflect the relative importance of direct use value, in this case the ecosystem service of recreational fishing (Table 1), for people who live close to the Daly River. Proportionately, however, people from southern Australia, far away from the river, were willing to pay about 60% of the total for the river's cultural importance compared with about 50% of those in the North who had capacity for direct use. Since ‘low income from irrigated agriculture’ had a negative sign and the medium level was not perceived as important, it would appear that respondents are not pressing for the amount of water extracted for irrigation to be increased but do think it should not be low, i.e. that Daly River water should be used for some irrigation to generate social benefits such as jobs.

**Table 4:** Monetary mean values of attributes (in AUS$) and 95% confidence intervals (CI) for all three samples.

	Australia		Northern Australia+		Southern Australia++	
	*Mean*	*CI*	*Mean*	*CI*	*Mean*	*CI*
Healthy floodplains medium	39	9–68	45	4–84	24	8–40
Healthy floodplains large	91	43–137	125	62–185	43	28–57
3-star fishing quality	100	52–145	131	53–207	53	48–58
4-star fishing quality	120	61–177	163	74–248	74	43–103
Waterholes in ok condition	116	60–170	98	30–164	91	73–108
Waterholes in good condition	161	88–231	207	91–320	103	85–120
Low income from irrigated agriculture	−74	−131–−19	−92	−181–−5	−52	−72–−32

+ respondents from the North included Darwin and the Daly River catchment.

++ respondents from the southern Australia were from Sydney.

### Aggregated Benefits for a Conservation Programme

The overall mean stated WTP for a Daly River conservation program was estimated at $298 per person ($161 for ‘waterholes in good condition’+$120 for ‘4-star fishing quality’+$91 for ‘large area of healthy floodplains’+($-74) for ‘low income from irrigated agriculture’), $403 for respondents from the North and $168 for respondents from southern Australia. About $21.4 million would be available if working individuals in the North would pay for a Daly River conservation programme (Table 5). If all Australian taxpayers would be targeted (e.g. via a tax), the potential value of a Daly River conservation budget is about $1.7 billion. These figures were extrapolated in a conventional way directly from the CE results. As discussed below, we also ‘anchored’ these figures to real market data and derived more realistic aggregate values (Table 5).

**Table 5:** Aggregate values of ecosystem services.

Approach	Australiaa)	Southern Australiab)	Northern Australiac)
*Conservative* +	$1 737 340 000 (11 000 000×0.53×$298)	$276 914 400 (3 110 000×0.53×$168)	$21 359 000 (100 000×0.53×$403)
*‘Anchored’*	$507 210 000 (11 000 000×0.53×$87)	$80 766 700 (3 110 000×0.53×$49)	$6 254 000 (100 000×0.53×$118)

a) The relevant population is 11 m employed Australians [69].

b) The relevant population is 110 000. There are about 150 000 people living in Darwin and the Daly River catchment. The labour force participation in the NT is 73.4% [70].

c) The relevant population is 3 110 000. Sydney has about 4.5 m citizens with a proportion of working aged people of 69.1% [69].

+ For the conservative approach it is assumed that 70% out of 67% would not pay (i.e. 47%) and that hence 53% would pay.

## Discussion

### Relative Value of Environmental Services

While we have demonstrated that distant and local tropical river water users are willing to pay for the ecosystem services that provide recreational values (high fishing quality), biodiversity values (healthy floodplains) and cultural values (good condition of waterholes for Aboriginal people), there remains some enthusiasm for irrigated agriculture which could hinder conservation initiatives. The benefit of the CE method, however, is that it can be used to assess the relative worth of attributes. The negative value for low agriculture is less than peoples’ WTP for the highest levels of the other three attributes, particularly for maintaining the quality of waterholes for Aboriginal people. Surprisingly, given the iconic status of the Daly River among recreational fishermen, the cultural value of the river was 25% higher than for high quality fishing and more than 40% higher than the biodiversity values. While it could be argued that high quality waterholes are unlikely to reduce fishing quality or the health of floodplains, the preference was nevertheless significantly higher for the cultural values over other uses, even among people in the North with direct access to the river.

### Translation of Willingness-to-pay into Financial Investment

Stated preference methods such as CE have gained acceptance as a valid means of quantifying environmental values around the world. In Australia CE has also become a valid tool among researchers to evaluate non-market aspects of Australia’s natural resources, with the first applied CE published in the mid-nineties [47], [48], [49]. Australian policy-makers, however, prefer revealed preference methods such as hedonic pricing and travel-cost methods over stated preference methods, as advocated “as a general rule” in the Best Practice Regulation Handbook [50]. However, non-market values cannot be assessed using revealed preference methods. Following the advice of the Office of Best Practice will therefore always lead to an underestimation of environmental values because the TEV can never be revealed, only indirect and direct use-values. Despite great methodological advances in eliciting TEV, empirical economic valuation that largely ignores non-use values continues to carry greater weight in Australia. One of the criticisms of WTP applications in environmental economics is that few questionnaires end with a request for real money and the relationship between hypothetical WTP for non-markets goods and the WTP in real money is poorly understood. While for private marketable goods the hypothetical WTP can be compared to the real price (known as an external validity test), it cannot easily be done for public goods such as ecosystem services [51]. However, Carlsson and Martinsson [52] did compare WTP results of a hypothetical and a real CE for environmental management valuation and concluded that there were no differences between them. We applied an external validity test to see whether or not hypothetical WTP differs from actual WTP.

A substantial number of respondents stated explicitly that they did not consider the monetary attribute when choosing among scenarios, which can lead to erroneous and biased results [37], [38]. Moreover, a separate analysis suggested that, for Aboriginal people, the northern rivers are beyond value in their current state [27] and hence for the Aboriginal sub-sample in this study (N = 37), the values elicited by the CE could be underestimated. In the case of the Daly River it is indeed unlikely, for many reasons, that there would be genuine enthusiasm to pay $1.7 billion in tax to retain the values of one northern river. However there is merit in comparing WTP estimates for individual attributes with the amounts that are actually spent as it is likely that the hypothetical WTP will be proportional to the actual amounts people have shown that they are prepared to pay for similar services.

The best market values available relate to fishing. While no government license fees are paid for fishing in public waters in the NT, the average for an annual permit for Australian states is $35 (Queensland dams ($35), Tasmania inland waters ($68.50), Western Australia freshwater angling ($30), New South Wales ($30), Victoria ($22). This is about a third of the hypothetical WTP (29%). The advantage of anchoring the fishing value, is that it can then be used to estimate the true WTP for the less tangible values for which no market exists. Thus the true WTP for a Daly River conservation programme can be calculated at $87 per person ($47 for ‘waterholes in good condition’: 0.29×$16+ $35 for ‘4-star fishing quality’ (the costs of a fishing permit)+$27 for ‘large area of healthy floodplains’: 0.29×$91+ ($-22) for ‘low income from irrigated agriculture’: 0.29×$(−74) ), at $118 for respondents from the North and at $49 for respondents from southern Australia. Based on these calculations, the amount that taxpayers in the northern NT would be willing to see invested in this service would be $6.3 million on the conservative assumption that 53% would be willing to pay (Table 5). Nationally the equivalent figure is about $507 million and for people in the South $80.8 million.

However, while licence fees may be used to support fishery management, they do not guarantee the quality of the fishing, so this empirical WTP estimate would almost certainly be exceeded were fishing quality assured. Even taking $35 as the real WTP estimate, the aggregate figures are still substantial. For the NT population for fishing alone the aggregate value was $2 million, which compares to an estimated $34.8 million spent on recreational fishing in the NT when last surveyed over ten years ago [53]. Of this 75% was spent by local residents ($26.1 million) – an average of $595 per year per fisher aged five years and above [54]. Spending $35 on an annual fishing pass, while not currently politically acceptable in the NT [55], constitutes <6% of annual expenditure per fisher ($595). Visitors accounted for 25% of the total expenditures ($8.7 million) – an average of $246 per visiting fisher [54]. A fishing permit of $35 only accounts for 14% of these expenditures. The aggregate value for 4 star fishing for people from southern Australia was $35 million.

This adjusted anchored amount that could be paid to maintain the biodiversity value of the Daly River ($27) is not unreasonable. Although visitors to protected areas in the Daly River catchment, which includes Katherine River Gorge in Nitmiluk National Park, currently pay nothing, an annual fee of $27 compares well with a fee of $25 levied from interstate and international visitors for each visit to the nearby Kakadu National Park [56]. Another reflection of the conservation value of the river is the amount currently invested in Aboriginal ranger groups, or that will be in the future. Currently ranger groups in the region receive government funding to manage weeds and reduce the number of feral animals and a further $37 million is to be invested in the region to reduce the incidence of wildfire, with the environmental benefit of reduced greenhouse gas emissions [57], following similar programs elsewhere [58]. The connection between the empirical market value and the hypothetical WTP estimate is even more difficult to calculate when considering the compensation required by respondents for low levels of irrigated agriculture should policy be directed towards improving cultural, fishing and conservation values of the Daly River. At one level this compensation is already paid by society through various forms of financial assistance provided to agriculture (total agricultural subsidies in Australia were $2.8 billion in 2008, [59]). For instance all primary producers in Australia are eligible for a tax rebate on diesel used in agricultural production (currently about 30% of wholesale prices [60] while much infrastructure in the form of roads and their maintenance, is primarily for the benefit of agricultural industries. Such costs are particularly high in the Daly River catchment where flooding is frequent and distances between farms large and are justified on the basis that they support an industry that provides jobs and with a high multiplier effect in local towns [30]. Although many farm enterprises may not be able to survive economically without subsidies, the willingness of Australian society to pay such subsidies has been demonstrated over many decades. The comparison of the amounts used to subsidize agriculture with the relative investments in fishing and conservation and the relative WTP could be a fruitful area of future research.

This is particularly true of Australians’ WTP for the cultural values of waterhole quality. There are no existing equivalents for the cultural value, which is one reason it is hardly ever considered in economic valuation and cost-benefit analyses. Indeed a recent study estimating the economic values of the Daly River catchment for Aboriginal people acknowledges that it had to exclude the trade-offs between productive and cultural values, although it did calculate the replacement value of the fish caught by Aboriginal people at $367 per person per year, of which it considered 90% at risk from agricultural development of the watershed [61]. The overall WTP for waterholes in good condition was more than half the total yet there is almost no evidence of investment by government in maintenance of this ecosystem service.

### Involving Indigenous Australians

Knowing the value of the river’s ecosystem services leads to practical considerations such as who should provide the environmental services and how they should be provided. A vast amount of land in northern Australia is owned and partly managed by Aboriginal people. This includes 15% of the Daly River catchment while a traditional Aboriginal connection remains with almost all of the land not formally owned. As noted by Mueller [62], among others, Aboriginal people are among the best suited to look after their traditional country (in both traditional and non-traditional ways). That they should be paid for their services has also been recognized by government with the Aboriginal ranger program. Overall there has been an investment of $40 m per year, until 2013, for different programs (Working on Country, Indigenous Rangers, Indigenous Protected Areas) [63]. However Aboriginal land management programs are designed to deliver services that are strictly environmental, with government programs drawing their budgets from agencies responsible for the environment. There is thus also pressure to show that government investment in such programs can demonstrate environmental benefits, even though such benefits can be extremely difficult to prove within the short time-frame of government funding cycles; environmental degradation has been slow and it is likely that any recovery will take at least as long. It has been argued that, regardless of the environmental impacts, the multiple benefits that both Aboriginal people, and Australian society more generally, derive from Aboriginal engagement in managing their traditional country warrants investment from government agencies responsible for health, education and justice among others [64]. Particularly large monetary savings are potentially available in health costs [65]. However, for Aboriginal people, there is good evidence that one of the primary motivations for involvement in natural resource management is to fulfill cultural obligations to look after their land, with environmental outcomes being a secondary benefit from that management [66], [67]. Thus, for government and other funding agencies, it is the cultural benefits that are subsidiary; Aboriginal land management groups are paid primarily for fire management and control of weeds and feral animals. A salient feature of the current study is that the different services were valued separately, and it would appear that people are willing to pay for both the environmental benefit of healthy floodplains and the cultural benefit of waterholes in good condition. Of particular interest here is that they are willing to pay about 40% more for the cultural values than the biodiversity values. A policy that reflects WTP would invest in actions that improve the cultural values of the Daly River, not just the environmental values. It would also be necessary to assess performance against this investment, metrics that can only be developed after extensive consultation with the traditional Aboriginal owners of the river.

### Conclusions

We have shown with an example from northern Australia that a choice experiment can be used to provide proxy values for a range of ecosystem services that we have then been able to ground in the market economy. Our approach can be used globally, and also for other ecosystem services for which users and providers are defined. In the case of the Daly River the ecosystem service valued most highly by both regional and distant users was the quality of waterholes important to Aboriginal people, a value for which there is no market and which cannot therefore be valued except through stated preferences. However, when this value is grounded against the market values for fishing, biodiversity and agricultural subsidies, it implies that government could justify substantial amounts to support the maintenance of such values. This value is in addition to such non-market values of the Daly River as its existence value (i.e. the value placed on knowing that the site exists for themselves and others in the current generation) and bequest value (i.e. the value of preserving the river for future generations). The conclusion that the cultural values of the river are important strongly supports the argument that these need to be considered in any water allocation plan for the Daly River in addition to, and separate from, other values, such as recreational and biodiversity values. While Aboriginal people in northern Australia are currently employed to maintain biodiversity and other ecosystem services [57], [68], they are not supported explicitly to retain the cultural values of landscapes. However, this research suggests that the wider Australian society is willing to pay for this service.
